# Silicon Cycling in Soils Revisited

**DOI:** 10.3390/plants10020295

**Published:** 2021-02-04

**Authors:** Jörg Schaller, Daniel Puppe, Danuta Kaczorek, Ruth Ellerbrock, Michael Sommer

**Affiliations:** 1 Leibniz Centre for Agricultural Landscape Research (ZALF), 15374 Müncheberg, Germany; daniel.puppe@zalf.de (D.P.); Danuta.Kaczorek@zalf.de (D.K.); rellerbrock@zalf.de (R.E.); sommer@zalf.de (M.S.); 2 Department of Soil Environment Sciences, Warsaw University of Life Sciences (SGGW), 02-776 Warsaw, Poland; 3 Institute of Environmental Science and Geography, University of Potsdam, 14476 Potsdam, Germany

**Keywords:** andosols, clay neoformation, crop yield, land use change, micro aggregate stability, phytoliths, sediments, silicon cycling, silicon extraction methods, silicon pore water speciation

## Abstract

Silicon (Si) speciation and availability in soils is highly important for ecosystem functioning, because Si is a beneficial element for plant growth. Si chemistry is highly complex compared to other elements in soils, because Si reaction rates are relatively slow and dependent on Si species. Consequently, we review the occurrence of different Si species in soil solution and their changes by polymerization, depolymerization, and condensation in relation to important soil processes. We show that an argumentation based on thermodynamic endmembers of Si dependent processes, as currently done, is often difficult, because some reactions such as mineral crystallization require months to years (sometimes even centuries or millennia). Furthermore, we give an overview of Si reactions in soil solution and the predominance of certain solid compounds, which is a neglected but important parameter controlling the availability, reactivity, and function of Si in soils. We further discuss the drivers of soil Si cycling and how humans interfere with these processes. The soil Si cycle is of major importance for ecosystem functioning; therefore, a deeper understanding of drivers of Si cycling (e.g., predominant speciation), human disturbances and the implication for important soil properties (water storage, nutrient availability, and micro aggregate stability) is of fundamental relevance.

## 1. Introduction

Silicon (Si) is the second most abundant element in the earth crust and occurs in a large number of minerals [1]. During the last decades, the interest in research on Si cycling in soils and sediments has strongly increased. Primary and secondary minerals, as well as biogenic silica (hydrated amorphous silica derived from organisms), can act as a source for silicic acid, i.e., the dissolved form of Si [2,3,4,5]. Weathering of specific minerals is also discussed as an important sink for CO_2_, because calcium carbonate formation from calcium silicates mobilizes silicic acid and binds CO_2_ [6,7]. In addition, phytoliths are discussed as a sink for organic carbon [8,9], which is potentially the carbon remaining from the protein template shaping the phytoliths [10].

Si fractions in soils comprise dissolved Si (in the form of monosilicic acid, polysilicic acid, or complexes of silicic acid and inorganic compounds) in the soil solution as a liquid phase [11], and the complexation of silicic acid with organic compounds was also suggested [11,12]. These dissolved Si species might adsorb to soil particles (especially Fe or Al oxides/hydroxides) [11,13,14]. The Si solid phase comprises different forms of amorphous Si (ASi): (1) biogenic amorphous Si (bASi, e.g., phytoliths, testate amoeba shells, diatom shells, silicified sponge spicules, radiolarian shells); and (2) minerogenic forms (silica nodules, silica included in pedogenic oxides such as iron oxides) [11,12]. The term “amorphous” had been introduced by mineralogists to describe non-crystalline phases when using X-ray diffractometry. However, in studies on silica chemistry in soils, the term “amorphous” is used to quantify Si, which can be extracted by defined reagents (Na_2_CO_3_, NaOH, TIRON etc.), i.e., without any statement about the (non-)crystallinity of the extracted phases. Other solid phases of Si are poorly crystalline and micro-crystalline forms (allophane, imogolite, opal-CT, chalcedony, and secondary quartz) [11]. Further Si phases in soils are crystalline forms in terms of primary silicates (e.g., quartz, feldspars, micas, olivines, pyroxenes, etc.) and secondary minerals (e.g., the different clay minerals) [11,12]. Crystobalite, for example, is a microcrystalline secondary quartz, which is a dominant mineral of volcanic rocks and has also been found in other soils [15]. Secondary quartz from opal can be found in cemented soil profiles [16]. Si is leached out, redistributed, or accumulated in soils. The process of desilication (Si leaching) is most pronounced in tropical soils [17], but can also occur in young soil from boreal zones [18]. Subsoil Si enrichments might be related to fragipan horizons’ development in temperate humid soils [19]. At the microscale, pure ASi coatings on soil minerals are commonly found in acidic soils [15,20]. Ongoing ASi infillings in soils’ pore system will lead to distinct SiO_2_ accumulations in soil horizons (duripan) [16] or massive sedimentary layers (silcrete) in semi-arid climates by repeated desiccation in the dry season [21,22]. Hardsetting soils (surface incrustations in semiarid soil) are also discussed as redistribution and precipitation of SiO_2_ [23].

Silicic acid occurring in soil solution might be taken up by plants, subsequently forming bASi [24]. Contents of bASi in plants are known to improve their resistance to cope with abiotic (e.g., drought, salinity, ultraviolet radiation, toxicants, or nutrient deficiency) [25,26,27,28,29] and biotic stress (e.g., herbivory, pests) [30,31]. All these effects led to an increasing interest of ecologists and agricultural scientists in the different Si pools in soils and Si availability in soils and sediments. However, silicic acid might not only be available for plant uptake, but also binds to (secondary) minerals [14], and is suggested to form secondary clay minerals if concentrations are near saturation [4].

Unfortunately, most reviews and studies on Si in soils argue with thermodynamic endmembers of corresponding reactions/processes or use aqueous pore water species, which are not stable for longer timespans under natural conditions, and thus corresponding results might therefore lead to potentially wrong assumptions. The problem why many assumptions are made on a less than ideal basis is the very low speed of specific reactions of Si in contrast to many other elements, making assumptions difficult when using methods originally made for analyses of elements with much faster reaction speeds. For example: (i) monosilicic acid requires several months for polymerization under low pH (pH ~4) and is much faster polymerized under higher pH, changing binding affinity to soil minerals [32]; (ii) silica-rich precipitations in soil solution are mostly considered as clay minerals despite the fact that mineral crystallization is a process that requires months to millennia, depending on the specific mineral [33,34].

Another reason for differences in the interpretation of study results seems to be the differing use of definitions. For example, in the fundamental work of Morse and Casey [34], it was stated that “sequential formation in time (paragenesis) of minerals in sediments frequently results in the formation of phases not predicted by equilibrium thermodynamics”, especially in regards to silica. However, a large number of studies argue with thermodynamic endmembers, e.g., a spontaneous formation of clay minerals from soil solution. Furthermore, many studies make general assumptions from short-term experiments using a certain species of silicic acid, which is not fully stable under the used conditions.

All these reasons hinder an overall process understanding of Si cycling in soils. Consequently, the aim of this review was to summarize the current knowledge and highlight research gaps regarding Si cycling in soil–plant systems. Firstly, we give an overview of the most important Si extraction methods, because this is the basis to understand the Si availability in soils (Section 2), followed by a section focusing on silicic acid and soluble silica species in soils and sediments (Section 3), because it is highly important to clarify the predominance of a certain Si species in soil solution to better assess potential effects on plants. Subsequently, we review biological controls on Si availability and Si cycling in terrestrial ecosystems (Section 4), followed by constraints on clay neoformation (Section 5). This section shows where existing literature is misleading, which is highly important to clarify because changes in occurrence patterns and processes alter Si effects in soils. In Section 6, we summarize knowledge on human impacts and global change effects on soil Si cycling, followed by a section on the importance of Si for crop production (Section 7). At the end of each section, we give a summarizing conclusion. The review ends with an outlook, where recommendations for future research are given (Section 8).

## 2. Si Extraction Methods

This section will provide a short overview of the most important currently used Si extraction methods and gained Si fractions (Table 1); it is important for all studies about Si cycling, availability, and function in plants and ecosystems to select the suitable extraction method. There are different protocols for Si extraction with pure water (i.e., water-soluble Si in soils). While Fox et al. [35] and Khalid and Silva [36] shook their samples, Schachtschabel and Heinemann [37] avoided shaking, because it was found that shaking increases Si mobilization due to mineral abrasion [38]. Extraction with CaCl_2_ is suggested to be a measure for readily “plant”-available Si. There are many different protocols for the use of CaCl_2_. It has been shown for paddy soils [39] that a 16 h extraction with CaCl_2_ is a much better predictor for plant-available Si [40] compared to, e.g., 1 h CaCl_2_ extraction [41]. Wu et al. [39] found that acetic acid as an extractant [42] (potentially extracting soluble Si and parts of the exchangeable Si) predicted plant-available Si in soils less accurately than the 16 h CaCl_2_ extraction. There are scant data comparing the method using NH_4_-acetate to extract Si [35] with other methods, but a comparable low relationship with plant-available Si (as for the acetic acid by Snyder [42]) can be assumed. Both extractants acetic acid and NH_4_-acetate potentially extract not only readily plant-available Si, but also some exchangeable Si [11]. NH_4_ citrate [43] potentially extracts soluble, exchangeable, and specifically adsorbed Si from soils [11]. Another method to be used for Si extraction is the Mehlich-III method, which is a standard for soil phosphorus extraction but also used to extract other elements such as Si [44]. This method extracts more Si than all other abovenamed methods [45], maybe even some parts of mineral Si. However, this method might be useful to quantify microbial-available Si in soil, because microbes are able to dissolve parts of minerals [46].

Oxalate, NaOH, Na_2_CO_3_ or Tiron as extracting agents aim for an extraction of amorphous silica, allophane, or imogolite-type minerals [11]. The oxalate method [47] seems to be able to extract Si bound in amorphous and poorly crystalline pedogenic oxides as well as oxy-hydroxides of Fe, Al and Mn, but certainly not all of them entirely (for more details see Sauer et al. [11]). Extraction with 0.5 M NaOH was found to dissolve not only ASi, allophane or imogolite, but also crystalline silicate. To reduce this negative side effect, Hashimoto and Jackson [48] reduced the reaction time to 2.5 min [11]. The method of Georgiadis et al. [49] using 0.2 M NaOH at room temperature almost completely extracted ASi and only small amounts of Si from crystalline compounds. However, the required extraction time of the method of Georgiadis et al. [49] to dissolve all ASi seems to depend on the condensation state (denoting the number of Si units bound to silicon atoms via oxygen) of ASi, which was already found to differ in plant materials [50], but might also differ in the soil. The Na_2_CO_3_ methods of DeMaster [51] and DeMaster [52] might underestimate the amounts of ASi in soils, but at the same time partially dissolve poorly crystalline minerals [49,53,54,55]. Kodama and Ross [56] found that Tiron dissolved less crystalline Si compared to the NaOH method, but dissolved similar amounts of ASi, allophane and imogolite.

Georgiadis et al. [57] developed a sequential extraction method for different fractions of soil Si. We will focus on the first six steps of this method. In step 1, soil samples are mixed with 0.01 M CaCl_2_ for 24 h to obtain readily plant-available Si. This part of the method might be comparable with the method of Haysom and Chapman [40]. Step 2 uses 0.01 M acetic acid to achieve a soil solution ratio of 1:10, which is then shaken for 24 h to obtain adsorbed Si. The authors claim that their method has a lower concentration of acetic acid compared to Snyder [42] to avoid potential Si release from other (more crystalline) sources. In Step 3, an oxidation of organic material is performed using H_2_O_2_ at 85 °C until the reaction is complete to obtain Si bound to organic matter. This fraction is missing in most other Si extraction methods [11], but might be very important for Si in soils. Step 4 uses 0.2 M NH_4_ oxalate and 0.2 M oxalic acid under ultraviolet light to extract Si occluded in pedogenic (hydr)oxides, and is comparable with the method of Schwertmann [47]. Step 5 is extracting biogenic ASi. For this, an aliquot of the samples from Step 4 (samples are divided for separate analyses in Step 5 and Step 6) is treated with sodium polytungstate according to Madella et al. [58] to obtain the biogenic ASi fraction from the soil. Then, this biogenic ASi fraction is dissolved using a 0.2 M NaOH solution to obtain biogenic ASi. A plateau correction is also suggested. This method is certainly not dissolving the share of crystalline Si as other NaOH extraction methods potentially do. Step 6 is extracting total ASi from another aliquot of Step 4 using a 0.2 M NaOH solution as in Step 5, but without the sodium polytungstate pretreatment. As an add on, the authors suggest calculating minerogenic ASi by subtracting the concentration of biogenic ASi from the total ASi concentration.

The sequential extraction method of Georgiadis et al. [57] is very promising. However, this method still has to be proven by comprehensive tests using different soils from different regions and ecosystems. As long as a widely accepted standard protocol is not established, we suggest using the CaCl_2_ method of Haysom and Chapman [40] for the extraction of “readily” plant-available Si, and the Tiron method of Kodama and Ross [56] or the Na_2_CO_3_ method of DeMaster [51] for the extraction of Si from ASi, allophane and imogolite. However, it is currently unknown how the condensation state of ASi in soils affects the extraction efficiency of the different methods.

## 3. Silicic Acid and Soluble Silica Species in Soils and Sediments

**Figure 1:**
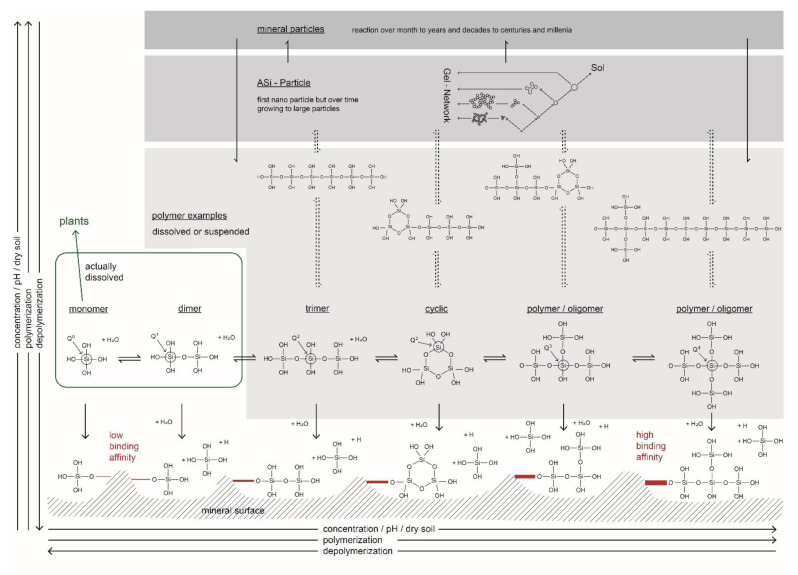
Scheme of: (i) polymerization/depolymerization of different species of silicic acid; (ii) precipitation to particulate ASi (first nano scale particles and later larger particle), crystallization and mineralization; (iii) dissolution of particulate to dissolved Si species; as well as (iv) the different condensation state. Dotted lines show potential reactions, for example of silicic acid speciation changes during polymerization/depolymerization. Differentation between actually dissolved species and species with higher polymerization can be performed by the molybdate method [64] or ultrafiltration (3 kDa) [65].

The predominance of a certain Si species in soil solution is highly important to better assess potential effects on plants in terms of availability and functioning. Therefore, we will review the existing literature on Si speciation in soil solution to draw an overall picture and reveal research gaps. There are abundant different species of Si in solution ranging from monomers to oligomers and with further increasing numbers of Si units to different forms of polymers, which tend towards gel formation and precipitation (Figure 1) [59,60]. Silicic acid with only one Si unit (monomer) is called monosilicic acid (orthosilicic acid). All condensates of silicic acids with more than one Si unit (dimers, trimers, tetramers, oligomers/polymers) are called polysilicic acids [61]. The solubility of silica in pure water at room temperature (limited by the amorphous phase) is about 100 ppm or ~1 mM, but increases at higher pH and higher temperatures [62,63]. Solutions of silicic acids become unstable with increasing degrees of condensation (from Q^0^, Q^1^, Q^2^, Q^3^ to Q^4^ groups, 0–4 denoting the number of Si units bound to silicon atoms via oxygen) tending to gel formation followed by precipitation (Figure 1) [59]. The differentiation between the different polymerization stages can be performed using the molybdate method which reacts and preferentially measures monomers and dimers forming a yellow-colored β-silicomolybdato complex [60], and is not that sensitive for higher polymerized species [64]. It was further shown that ultrafiltration (3 kDa) is in accordance with the molybdate method [65]. The data of Audsley and Aveston [66] also suggest that also ultracentrifugation could be used for the separation between the different polymerization states of silicic acid. However, to differentiate between actually dissolved silicic acid (monomer and dimer) and polysilicic acid, we suggest using more than 50,000× *g*. Factors controlling condensation are concentration, temperature, and pH, as well as the presence of other ions, molecules and polymers [63]. It was shown by Dietzel [67] that polysilicic acid is mobilized during the dissolution of Si-rich solids. Dissolution of different forms of silica (amorphous silica, silica gel, opal, volcanic glass, quartz or other minerals) produce monosilicic acid until equilibrium is reached [63]. However, at early stages of dissolution, the presence of polymeric Si species might account for ~50 mol % of total dissolved silica [67]. These polymers converted over time to monomers in an experiment at pH of 3 (buffered by sulfuric acid) [67]. Using natural water, the depolymerization to monomers took days to ~1 year for natural acidic water (pH 3 to 5.5), ~1 day for water rich in salt (brine or seawater with a pH between 6.1 and 8.2), less than one hour to ~1 day for river and ground water (pH from 6.5 to 8.3), and less than one minute for alkaline water (pH between 9.3 and 12.9) [67]. Depolymerization was also shown to be affected by ions and anions in solution [68]. The depolymerization rate decreased for ions in the order (1) Na^+^, K^+^; (2) Mn^2+^, Mg^2+^, Ca^2+^, Sr^2+^; to (3) Zn^2+^, Ni^2+^, Ce^3+^, Cu^2+^, and increased for anions in the order NO_3_^−^, HCO_3_^−^, C1^−^, SO_4_^2−^, whereas HPO_4_^2−^ caused a decrease [68]. However, all these depolymerization experiments by Dietzel [67] and Dietzel and Usdowski [68] were conducted at concentrations far below saturation. Hence, if the concentration of silicic acid is lower or far below saturation, silicic acid will depolymerize.

With increasing silicic acid concentrations, polymerization of the metastable monosilicic acid to polysilicic acid in terms of dimers and short linear oligomers proceeds (Figure 1) [69]. Polymerization and subsequent precipitation were found to be accelerated with increasing pH from 3 to 6 [69]. It was suggested that nanocolloids are relatively stable at low pH and ionic strength, because polymerization also increased with ionic strength [69]. The same trend was suggested for soil solution of acidic forest soils (pH ~4) with increasing shares of polymerized silicic acid with increasing concentrations and pH [65]. Another study showed that monosilicic acid requires several months for polymerization under low pH (pH ~4), and is polymerized much faster under higher pH [32]. Modelling suggested silicic nanocolloids as potentially being an important species under low pH and neutral conditions [70]. The presence of other elements, for example metals such as lead, copper or cadmium, in soil solution was also shown to be a driver for polymerization and precipitation [71], but this process might also happen in the presence of other elements. This finding suggests that silicic acid is a main control on ion concentration in soils. The occurrence and concentration of silicic acid in solution is regulated by: (i) polymerization/depolymerization; (ii) complexation of silicic acid with inorganic and organic ligands; but also by (iii) adsorption/desorption to or from mineral or organic surfaces [72]. The binding strength of monosilicic acid to, e.g., goethite, at short reaction times is low [73]. Adsorption of polysilicic acid to mineral surfaces is much faster (some minutes) compared to sorption of monosilicic acid (sorption within weeks), because polysilicic acid has a higher binding affinity than monosilicic acid [61]. During adsorption of polysilicic acid to mineral surfaces, one part of the polysilicic acid is bound to the mineral surface within a short time, whereas the other part of polysilicic acid is decomposed to monosilicic acid and released to solution (Figure 1) [61]. The adsorption of monosilicic acid to soil minerals increases from hematite, goethite, magnetite, lepidocrocite, akaganeite, feroxyhyte, ferrihydrite, amorphous iron hydroxide, to gibbsite [61]. The adsorption of silicic acid to mineral surfaces is a reversible process [61]. Polysilicic acids seem to be metastable, not only in solution but also when bound/complexed to the surface of minerals, where depolymerization also occurs, releasing monosilicic acid to soil solution (Figure 1) [61]. Monosilicic acid mobilization from polysilicic acid sorption to mineral surfaces is decreasing with decreasing pH [61]. Monosilicic acid is more abundant in natural systems compared to polysilicic acid, because monosilicic acid adsorption is much slower (Figure 1) [61]. Polysilicic acid is stable under alkaline conditions [61]. However, under slightly alkaline conditions, the adsorption of monosilicic acid seems to be favored [61].

In periods where soil water is scarce due to plant uptake or soil evaporation, the concentration of silicic acid increases due to water loss, potentially leading to concentrations above the maximum of Si solubility, resulting in Si precipitation [60,74]. Therefore, condensation/polymerization of silicic acid occurs (Figure 1). In each condensation reaction of silicic acid, one water molecule is set free (Figure 1) [63]. During polymerization at high Si concentrations, condensation nuclei are formed followed by the formation of nano-particles as suspension (Figure 1) [60,63]. This is followed by particle size growth driven by the dissolution of smaller particles and the redeposition of the dissolved species on the surface of the growing particle (Ostwald ripening) [63]. During a longer timespan (month to years or decades, or even centuries or millennia) a mineral formation by crystallization might occur (see above).

There are some articles suggesting a complexation of silicic acid with bi- and trivalent cations (e.g., Fe at pH > 3; Ca, Mg, Cu, Cd, Pb, Al) [75,76,77,78,79]. However, most knowledge on silicic acid reactivity is gained from experiments using pure water or performed under artificial conditions, which are not likely to occur in soil and sediment pore waters. Hence, it is currently unknown whether these complexes form in natural soil solution, and under which conditions and how they interact with each other. However, if these complexes are formed, they will certainly influence the behavior and availability of silicic acid in soils.

Although most complexation experiments did not reflect on the complex soil pore water system, they indicated: (i) the abundance of polymeric Si species at early stages of soil mineral dissolution; (ii) the depolymerization to monosilicic acid if the Si concentration is far below saturation; while (iii) polymerization will increase if the Si concentration is near saturation. The polymerization process is also positively related to ionic strength and pH. However, regarding more complex systems such as soil or sediment pore water, where the interaction of different cations and anions at different concentrations of silicic acid, ionic strength and pH potentially affect silicic acid polymerization, only a few studies have been performed so far. Monosilicic acid is the most abundant Si species in natural systems compared to polysilicic acid, because monosilicic acid adsorption to mineral surfaces is several orders of magnitude slower compared to adsorption of polysilicic acid. Most importantly, the adsorption of silicic acid to minerals surfaces is a reversible process. During the precipitation of polysilicic acid to ASi particles, nanoparticles occur first followed by particle growth leading to large ASi particles.

## 4. Biological Controls on Si Availability and Si Cycling in Terrestrial Ecosystems

### 4.1. Biogenic Silica Pools in Soils and Their Relevance for Si Cycling

**Figure 2:**
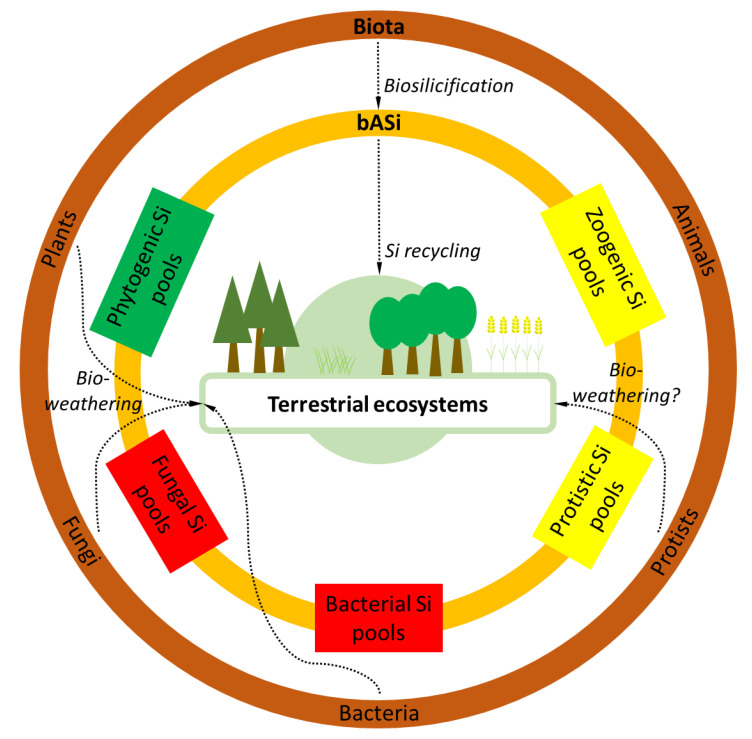
Schematic overview of the role of biota, biosilicification, and bASi pools in Si cycles of terrestrial ecosystems. The different colors of boxes of bASi pools indicate the corresponding level of knowledge on corresponding Si pool quantities (green, numerous studies available; yellow, only few studies available; red, no studies available).

Numerous organisms have evolutionarily adapted to use Si for the synthesis of siliceous structures, in a process called biosilicification. In fact, biosilicification occurs in prokaryotes as well as in eukaryotes (reviewed by Ehrlich et al. [80]). These organisms use monomeric silicic acid (H_4_SiO_4_) for the synthesis of hydrated amorphous silica (SiO_2_·*n*H_2_O), which is called biogenic amorphous silica (bASi). Based on their origin, bASi structures and residues in soils represent bASi pools that can be distinguished as follows: (i) bacterial bASi (formed in bacteria); (ii) fungal bASi (formed in fungi); (iii) phytogenic bASi (formed in plants); (iv) zoogenic bASi (formed in animals); and (v) protistic bASi (formed in protists) [81] (Figure 2).

bASi plays a key role in the link between global Si and carbon cycles; bASi controls Si fluxes from terrestrial to aquatic ecosystems because it is in general much more soluble compared to silicate minerals [18,82,83,84,85]. Due to the fact that marine diatom reproduction strongly depends on Si bioavailability (Si is needed for diatom frustule formation), these Si fluxes control marine diatom production on a global scale. Marine diatoms, in turn, are able to fix large quantities of carbon dioxide via photosynthesis, because up to 54% of the biomass in the oceans is represented by these unicellular organisms [86,87].

Furthermore, various favorable effects of Si accumulation in plants have been revealed, i.e., increased plant growth and resistance against abiotic (e.g., drought) and biotic (e.g., fungal infections) stresses, which is why Si is considered as a beneficial substance for the majority of higher plants [24,88,89]. Thus, bioavailability of Si in soils is crucial and bASi pools, especially the phytogenic ones, play an important role as source of bioavailable or readily or plant-available Si (H_4_SiO_4_), a factor that is of special importance for agricultural soil–plant systems [90,91,92]. However, due to intensified land use (agriculture and forestry), humans directly influence Si cycling on a global scale. Si exports via crop harvesting and increased erosion rates generally lead to a loss of bASi in agricultural plant-soil systems, and thus deplete plant-available Si in agricultural soils (known as anthropogenic desilication) [93,94,95]. Aside from climate change, a growing global population, and decreasing resources [96,97,98], anthropogenic desilication might be one of the major challenges for agriculture in the 21st century. In agricultural plant–soil systems, bASi losses of up to 100–500 kg Si ha^−1^ (depending on crops) occur by crop harvesting year by year [99,100]. About 35% of total phytogenic bASi has accumulated in field crops on a global scale, and this proportion is going to increase with increased agricultural production within the next decades [101].

This clearly emphasizes the need for a profound understanding of anthropogenic desilication and its prevention, especially in agricultural plant–soil systems. Thus, we urgently need detailed research on biosilicification by different organisms, corresponding bASi pool quantities, and physicochemical properties of bASi structures, because these properties control bASi solubility, and thus Si release rates [102,103,104]. While corresponding research on phytogenic bASi has been performed for decades now (see Section 4.2), studies on other bASi pools are still rare (see Section 4.3).

### 4.2. Plants and Phytogenic Silica

**Figure 3:**
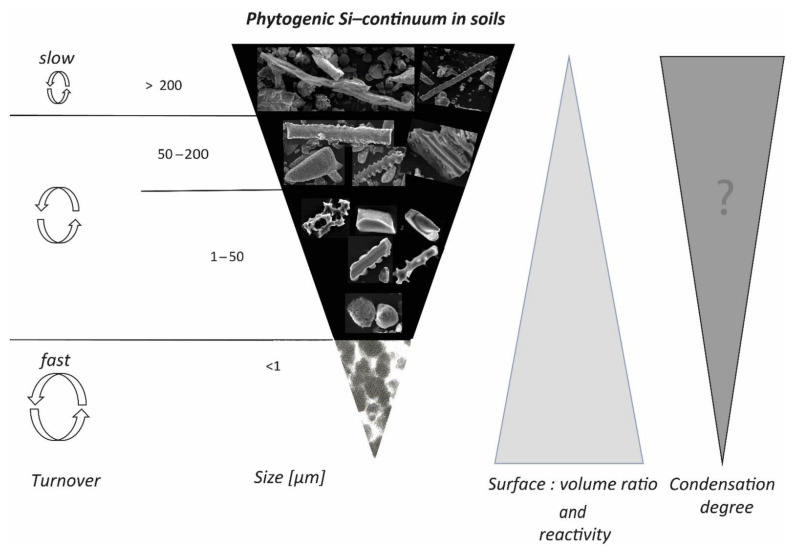
Conceptual model of the phytogenic Si continuum in soils.

Phytogenic silica and phytoliths (i.e., the siliceous structures precipitated in plants) are mainly made of SiO_2_·*n*H_2_O, but also contain organic matter and various elements such as aluminum, calcium, iron, manganese, and phosphorus [105,106,107]. Phytogenic silica can be found in living plants within cells (i.e., in the cell wall and the cell lumen), forming relatively stable, recognizable phytoliths, or in intercellular spaces and extracellular (cuticular) layers forming relatively fragile silica structures [108,109]. Phytogenic Si can be found in almost any plant organ, e.g., in leaves and stems as well as in roots [110,111]. Phytoliths can be frequently found in most soils and show a specific morphology that can be used for the taxonomic identification of plants [108,109,112]. However, the fragile and small silica structures (<5 µm) are usually not covered by standard phytolith extraction methods [113], but have been found to potentially represent the biggest and most reactive phytogenic bASi pool in soils [114]. We ascribe differences in phytolith stability to physicochemical properties (e.g., soil pH, Si availability, and plant Si demand) as well as the degree of silica condensation [50], i.e., silica in recognizable phytoliths shows a higher degree of condensation than silica in fragile forms such as the Si double layer, which is faster dissolved during organic matter decomposition [115] (Figure 3, see also Section 4.4). In this context, the degree of silica condensation might be also influenced by the location of phytolith formation (e.g., cell wall phytoliths seem to be less stable than lumen phytoliths, see Hodson [116]).

Si contents vary considerably between plant species, with values ranging from about 0.1–10% Si per dry mass [117]. Based on their Si content, plants have been divided into three groups: (i) non-accumulators or excluders (Si content per dry mass <0.5%); (ii) intermediate accumulators (Si content per dry mass 0.5–1%); and (iii) accumulators (Si content per dry mass >1%) [118]. Field crops, especially cereal grasses of the family Poaceae (or Gramineae), are known as Si accumulators [117]. Si absorption by plants is controlled by specific influx (called Lsi1 and Lsi6) and efflux (called Lsi2) channels, which have been found especially in crops such as rice (*Oryza sativa*), wheat (*Triticum aestivum*), or sorghum (*Sorghum bicolor*) (see Ma and Yamaji [119] for a detailed review). However, it should be kept in mind that the mechanisms behind the uptake, transport, and accumulation of Si in plants (active vs. passive Si transport) as well as Si-induced plant resistance (mode of action of Si in plants) are still not fully understood, and thus are under controversial discussion (see, e.g., Frick et al. [110], Coskun et al. [120], Exley et al. [121], and Exley [122]). For example, it was found that plant functional groups strongly affect Si stocks in aboveground biomass, with grasses increasing and legumes decreasing the aboveground biomass Si stocks [123]. It was further suggested that the Si availability in soils in relation to the calcium availability might control the dominance shifts between grasses (Si accumulator plants with plant protection based on Si) and legumes (calcium accumulation plants with plant protection based on calcium) [124].

Although Si is very abundant in the Earth’s crust (>90 vol.% consists of SiO_2_ and silicates), Si bioavailability is often limited in soils, because Si is: (i) leached as a result of rainfall and irrigation, especially in agricultural soils [125]; and (ii) bound to the soil minerals surface and competes for binding sites there with, e.g., phosphorus, and organic carbon [126,127]. Soils with a low Si bioavailability generally can be characterized as highly weathered, leached, acidic, and low in base saturation. Such conditions are typical for many soils in (sub)tropical regions, where two well-known Si accumulators are grown, i.e., rice (*Oryza sativa*) and sugarcane (*Saccharum officinarum*), commonly making use of Si fertilizers (see Datnoff et al. [128] and references therein). For the determination of bioavailable Si, several extraction methods (e.g., calcium chloride, acetate/acetic acid, or citrate extractions) have been developed (see Section 2). In agricultural soils, especially, the influence of: (i) adsorption/desorption reactions; (ii) leaching of Si from soils; (iii) bASi pools; and (iv) soil pH on Si bioavailability have been the focus of attention, but still need a deeper understanding in general [90]. Furthermore, it was suggested that plants can actively increase Si bioavailability in soils by increasing soil weathering process in the rhizosphere [129,130]. However, it is very likely that the uptake of Si from soil solution into plants by itself is already reducing the concentration of silicic acid in soils, and thus potentially increasing Si mobilization driven by an increased concentration gradient.

Si uptake and storage in plants has been analyzed for several ecosystems. Regarding natural ecosystems, Si storage in aboveground vegetation has been reported, e.g., for the Great Plains (short grass steppe and tall grass prairie: 22–67 kg Si ha^−1^, Blecker et al. [131]), the tropical humid grass savanna (tall grass *Loudetia simplex*: 33 kg Si ha^−1^, Alexandre et al. [132]), and forested biogeosystems (beech forest: 83 kg Si ha^−1^, Sommer et al. [2]; beech–fir forest: 180 kg Si ha^−1^, pine forest: 90 kg Si ha^−1^, Bartoli [133]). The amounts of Si uptake per year, i.e., annual biosilicification rates, have been reported for, e.g., beech (35 kg Si ha^−1^ yr^−1^, Sommer et al. [2]), beech–fir (26 kg Si ha^−1^ yr^−1^), and pine (8 kg Si ha^−1^ yr^−1^, Bartoli [133]), Douglas fir (31 kg Si ha^−1^ yr^−1^), Norway spruce (44 kg Si ha^−1^ yr^−1^), black pine (2 kg Si ha^−1^ yr^−1^), European beech (23 kg Si ha^−1^ yr^−1^), and oak forests (19 kg Si ha^−1^ yr^−1^, Cornelis et al. [134]). For agricultural sites, Si uptake has been reported for, e.g., wheat (20–113 kg Si ha^−1^ yr^−1^, Keller et al. [135]), rice (270–500 kg Si ha^−1^ yr^−1^, Keller et al. [135]; 230–470 kg Si ha^−1^ yr^−1^, Savant et al. [136]), and sugarcane (379 kg Si ha^−1^ yr^−1^, Savant et al. [137]). These data impressively indicate the potential of crops for Si accumulation driven by relatively high biomasses as well as Si concentrations. In contrast to natural ecosystems, where bASi is recycled in great amounts, agricultural sites are subject to high Si exports by harvest (the Si uptake rates above can be assumed to equal annual Si exports) year by year with implications for Si bioavailability in agricultural soils (anthropogenic desilication), and thus Si cycling in agricultural soil–plant systems (Struyf et al. [93], Vandevenne et al. [94], Vandevenne et al. [95]; see also Section 7).

### 4.3. Further Organisms and Corresponding bASi Pools

Regarding protozoic bASi, several studies have been conducted (reviewed by Puppe [81]). Protozoic silica in soils is mainly synthesized by testate amoebae, which form a polyphyletic group of unicellular eukaryotes (protists) with a shell (or test) ranging between about 5–300 µm. Testate amoebae can be assigned to two supergroups: (i) the Amorphea, including the order Arcellinida; and (ii) TSAR, including the order Euglyphida [138,139]. The order Arcellinida includes testate amoebae with lobose pseudopodia and shells made by secretion (autogenous shells), agglutination of foreign materials collected in the environment (xenogenous shells), or a combination of secretion and agglutination. The order Euglyphida includes testate amoebae with filose pseudopodia, and almost all extant species in this order are characterized by siliceous shells made up of self-synthesized silica platelets, the so-called idiosomes. Research on protozoic silica has been focused on species in the order Euglyphida, although a few taxa (i.e., *Lesquereusia*, *Netzelia*, and *Quadrulella*) with autogenous siliceous shells can also be found in the order Arcellinida. The silica platelets of testate amoebae are formed in so-called silica deposition vesicles (SDVs) in the cell cytoplasm and deposited on the cell surface by exocytosis, where they are finally bound together by organic cement [140,141,142,143,144].

At the beginning of the 21st century, the potential of protozoic silica for Si cycling was mentioned in some publications [18,145,146,147]. Shortly thereafter, Aoki et al. [148] were the first to quantify bASi in the shells of different testate amoeba taxa in the order Euglyphida. Based on these results, they further quantified protozoic silica pools in pine–oak forest soil in Japan, and, using data of annual mean population densities from the literature, calculated annual biosilicification rates of living testate amoebae. In doing so, Aoki et al. [148] showed annual biosilicification of idiosomic testate amoebae to be comparable to silica released by trees via litter fall, and thus testate amoebae to potentially be as important for global Si cycling as trees. Although this potential was recognized by some authors [80,84,85,149,150], it took some more years until the quantification of protozoic silica pools and annual bio-silicification of testate amoebae and implications for Si cycling became the focus of attention of several researchers [2,151,152]. These studies clearly showed that biosilicification by testate amoebae has to be considered in analyses of Si cycling in terrestrial biogeosystems, because Si fixation in testate amoeba shells is comparable to or even can exceed the amounts of Si absorbed by trees year by year [81,153].

Aside from testate amoebae, terrestrial diatoms have been found to play a role in Si cycles of some ecosystems [114,152,154]. Indeed, the role of protists (e.g., testate amoebae, diatoms) in biogeochemical cycles gained broad attention in the scientific community. For example, Geisen et al. [155] published a synthesis of research gaps in the field of soil protistology, which should be a priority focus in future studies. They proposed 30 key questions, covering a broad range of areas including evolution, phylogenetics, functional ecology, macroecology, paleoecology, and methodologies to identify “hot topics” for the focus of future research. Interestingly, the most important question for future research was: What is the importance of soil protists in biogeochemical cycling? To answer this question, we urgently need more studies on biosilicification by unicellular organisms and their impact on Si cycling in terrestrial ecosystems. Regarding sediments, other protists such as Heliozoa (inhabiting freshwater and marine environments) and Radiolaria (inhabiting marine environments only) should also be in the focus of attention, because although we do know about the mechanisms of biosilicification in these organisms [143], there are still no quantitative data on corresponding Si pools [145].

In addition, we urgently need more research on bASi synthesized by sponges, fungi, and bacteria. While there are few studies on zoogenic Si pools (sponge spicules) and their role in Si cycling in terrestrial biogeosystems [114,154] and freshwater lakes [156], there are no studies on fungal and bacterial Si pools in terrestrial ecosystems. At least, we know that some bacteria (e.g., *Proteus mirabilis*) and fungi are able to accumulate Si within their cells, as shown by Ehrlich et al. [80] and references therein. Furthermore, these organisms can enhance the dissolution of amorphous and crystalline silica (bio-leaching or bio-weathering, reviewed by Ehrlich et al. [80]), e.g., by the release of acidic metabolites, a process that is also known from plant roots (bio-weathering in the rhizosphere, see, e.g., Gattullo et al. [130]). There are also some hints that bio-weathering might play a role in diatoms [80,157], but there are no studies on bio-weathering by other Si-accumulating protists such as testate amoebae available as far as we know. This clearly shows that we are still at the beginning of understanding the importance of biota for Si cycling, especially regarding microscopic organisms and their role in biogeochemical Si cycles (Figure 2).

### 4.4. The Phytogenic Si Continuum in Soils

In soils and sediments, phytogenic Si can occur either within plant fragments or within the groundmass [158]. The distribution of phytogenic Si in soils is rather variable [113,145], although the highest contents in undisturbed soils were suggested for surface horizons [159,160,161]. The size of Si precipitates in plants ranges from 100 nm [161] to 1 mm [162]. In soils, phytoliths Ø > 5 μm (e.g., elongate and bilobate phytoliths, trichomes), phytoliths Ø < 5 μm, and small-scale (<1 µm) phytogenic Si structures exist. Phytoliths larger than 5 μm represent only about 16% of total Si contents of plant materials of *Calamagrostis epigejos* and *Phragmites australis* (Poaceae) [114]. Wilding and Drees [163] showed that about 72% of leaf phytoliths of American beech (*Fagus grandifolia*) are smaller than 5 μm. These findings clearly point to the potential significance of phytogenic Si < 5 μm for Si cycling in general. The same holds true for weathered phytoliths, representing another large and highly reactive Si pool in soils [113,114]. Phytolith contents in most soil horizons are in the range between 0.01% and 3%, but can be even larger [2,113,114,164,165]. Total phytogenic Si in soils must be even higher, because phytolith analysis is normally restricted to silt-sized particles and discards the fraction <5 µm during the separation procedure. Single sedimentary soil layers (in colluvial soils) might show up to 90 wt.% SiO_2_ in the fine earth, almost exclusively derived from phytoliths [166]. It has been shown that phytoliths are characterized by a variable solubility in soils. The factors which control phytolith dissolution in soils are: (i) phytolith properties (i.e., specific surface area, aluminium (Al) content, condensation state, age, rate of organic matter biodegradation); and (ii) soil properties (i.e., soil pH and soil buffering capacity) [102,104,167,168,169,170,171]. Phytolith properties are highly variable and seem to depend mainly on phytolith morphotypes (i.e., phytolith geometry), although some studies also ascribe differences in phytolith dissolution to phytolith origin [2,172]. Grass phytoliths appeared to be less soluble compared to tree phytoliths [173]. Smaller phytoliths in soil profiles are also subject to translocation processes, especially driven by bioturbation and percolation [113,172,174,175]. Phytoliths represent a huge pool of relatively soluble silica in terrestrial ecosystems, and thus one of the main sources of Si in soil pore waters and aqueous ecosystems [84].

The previous classification of phytoliths applies only to well-developed and well-recognizable forms with specific shapes. There is no definition of phytoliths covering all different forms of phytogenic Si in soils. Therefore, we would like to propose a new concept for the presence of phytogenic Si in soils, i.e., a model of a “phytogenic Si continuum in soils” (Figure 3). This model is not confined to phytolithic Si, which is constantly transformed in the soil environment driven by soil and phytolith properties. Consequently, at any given time there is a continuum of many different forms of phytogenic Si at different stages of decomposition/dissolution in soils, ranging from large to small and from rapidly weathering to relatively stable phases—likewise, phytoliths. The phytogenic Si continuum seems to be a continuous function in the soil environment, mainly depending on the size, specific surface area, or degree of condensation of phytogenic Si.

For example, the Si double layer as a phytogenic Si form with a potentially lower condensation state and lower thickness of particles may dissolve faster into polysilicic acid. In general, the following can be stated as a rule of thumb: the lower the condensation state, the higher the specific surface area of phytogenic Si (including phytoliths), and the smaller the particles, the higher the potential dissolution rate. However, it remains unclear whether the condensation state or the surface area-to-volume ratio of phytogenic Si particles or the particle size is the more important factor for susceptibility to dissolution. We feel that research on these issues is urgent and of high relevance, especially when considering the significance of phytogenic Si for Si cycling in terrestrial biogeosystems.

**Figure 4:**
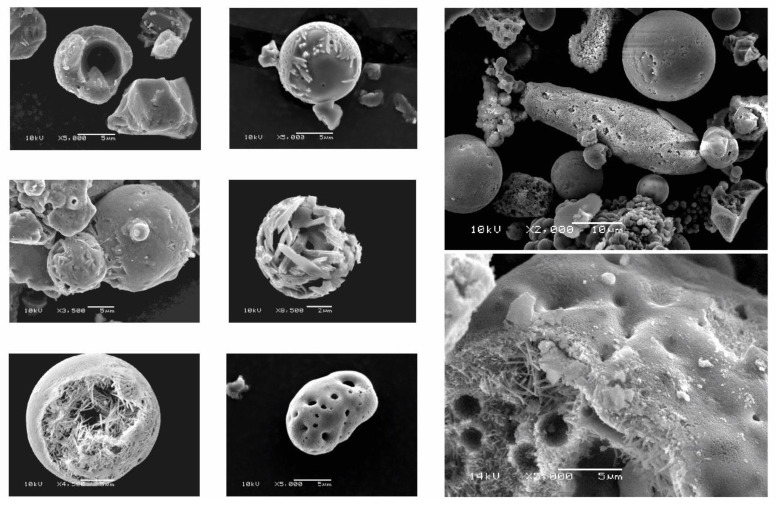
Examples of phytolith-like amorphous Al–Si compounds originating from fly ash.

In some soils, “strange” spherical phytolith-like structures can be found (Figure 4). These structures are amorphous Al–Si compounds, which indicate amorphous–crystalline transitions and originate from fly ash [176]. To avoid a confusion of these structures with phytoliths, care must be taken in phytolith analyses.

**Figure 5:**
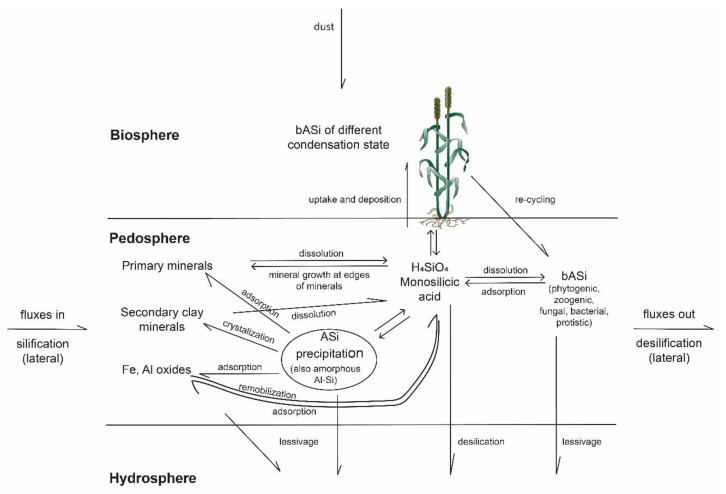
Conceptual model of Si fluxes and pools in the soil–plant continuum, modified after Cornelis and Delvaux [4].

On a global scale, plants in terrestrial ecosystems cycle about 60–200 T mol Si per year, indicating their significance for Si cycling in general [84]. Plant Si cycling includes the uptake of silicic acid by plant roots, accumulation of amorphous silica in below- and aboveground biomass, litterfall, and litter or organic matter decomposition [177,178,179,180], where silicic acid is mobilized again [181] (Figure 5). In this context, an important parameter affecting Si mobilization is the Si condensation state [50], which tends to be higher for raised platform phytoliths compared to, e.g., the double Si layer [115]. Other factors controlling phytogenic Si dissolution in soils are specific surface area, aluminium (Al) content, age, rate of organic matter biodegradation, soil pH, and soil buffering capacity. Due to the fact that phytoliths do not cover all forms of phytogenic Si in soils, we suggest a new conceptual model of a “phytogenic Si continuum in soils” (Figure 3). Furthermore, studies on bASi-synthesizing organisms other than plants are urgently needed. Currently, we have no idea about global annual Si cycling rates of, e.g., protists (testate amoebae, diatoms) in terrestrial ecosystems, because estimations of global Si cycling by biota “only” consider Si cycling by vegetation. In his review of studies on protozoic silica, Puppe [81] emphasized that it is very likely that testate amoebae are potential key players in the Si cycle of terrestrial ecosystems because: (i) annual biosilicification rates of idiosomic testate amoebae are comparable to or even exceed annual Si uptake rates of trees; and (ii) it is most likely that total protozoic Si pools (considering not only intact shells but also single idiosomes) are much bigger than stated in publications, however, because it can be assumed that idiosomes most likely can be as stable as phytoliths, and thus are well preserved in soils. Thus, it would not be surprising if total protozoic Si pool quantities (shells plus single idiosomes) would be found to be equal phytogenic Si pool quantities in soils.

## 5. Constraints on Clay Neoformation

The exact form of precipitates (amorphous or crystalline) of Si from soil solution is important, because the predominance of a specific form determines the availability and function of Si in soil toward plants. The main difference in terms of the availability and function of Si in soil is between crystalline precipitates such as clay minerals and amorphous precipitates such as ASi. This section will focus on clay neoformation, but not on clay reorganization/modification/transformation. Clays are the main component of most soils. The problem with the term “clay neoformation” is that it is used in two different ways: (i) to describe a size fraction (clay fraction: particle size < 2 µm); and (ii) to describe the formation of new clay minerals. However, the usage of the term “clay neoformation” is not always clear, because both meanings are mixed up or “clay neoformation” is even used incorrectly (clay mineral instead of clay fraction) in some studies. Thus, researchers should define whether “clay neoformation” is used to describe a size fraction or in terms of clay mineral formation in their article.

Clay minerals are made of two-layer structures, such as kaolinite or three-layer structures, e.g., montmorillonite or vermiculite. Each of these different clay minerals consists of a certain element stoichiometry. The formation of such clay minerals from soil solution is pH-dependent. For instance, the formation of kaolinite from a solution containing 6–12 mg/L Al_2_O_3_ and 15–40 mg L^−1^ H_4_SiO_4_ at a pH range from 4.5 to 5.3 requires several months [182,183]. These studies clearly showed that such clay minerals do not form instantly, but need at least months for the formation of crystal structures. Other clay minerals, such as smectite, require high amounts of Si, Al and Mg [184], while for the formation of others, the presence of elements such as K and Ca, among others, are needed. On one hand, temperature and element concentration in soil solution controls mineral dissolution and precipitation. On the other hand, the element concentration in soil solution depends on soil water content, water flow, pH, biological factors (see above), soil gases, and, again, temperature. As shown in the paragraph before, the Al solubility at a pH range from ~5 to ~7 is much lower as compared to that of silicic acid [185], decreasing the possibility of aluminosilicate formation (Figure 5). At soil pH values below 5 and low silicic acid contents, the availability of Si and Al might be in the same range such that an aluminosilicate precipitation can be assumed [4], resulting in the potential formation of smectite or kaolinite [186] (as the thermodynamic endmembers) over a period of weeks to years or even millennia. Lowe [187] suggested that “2:1 clays” form coevally with opaline silica (ASi) under excess “silica” (silicic acid) availability. He also suggested that the formation of opaline silica (ASi) is favored by water loss due to evapotranspiration by plants. In his work, Lowe [187] proposed that opaline silica and cristobalite form from silica hydrogel (polymerization of silicic acid). However, secondary quartz can also form from ASi or opaline silica [15] (Figure 5).

Most literature on “clay neoformation” exists for volcanic ash soils (classified as andosols or andisols) [188,189]. These soils occur in the surroundings of active or recently extinct volcanos [190], and commonly have a soil pH ranging from 4.8 to 6.9 [191]. Due to their high water-holding capacity and nutrient supply, these soils contribute significantly to wood and food production, despite their relatively low occurrence (<1%) on the global scale [190]. The main minerals of andosols are allophane and imogolite [192]. These minerals derived from 5000 to 10,000 year old ashes [192]. Volcanic glasses are dominant in the coarse fraction of andisols [193]. This glass material, with its porous nature and high content of allophane minerals, is known for its high water-holding capacity [194,195] and weathers quickly to a colloidal fraction. Another dominant share of andosols/andisols might be amorphous Si (ASi) [196]. The glass material fraction can be described as amorphous or nanocrystalline (allophane, ferrihydrite or imogolite) [197,198]. There is a further classification of andosols into sil-andic/non-allophane (rich in Si and poor in Al availability) and alu-andic/allophane (poor in Si and rich in Al availability with soil pH < 4.5 or free Al chelated with organic matter) andosols [191,199]. The availability of Al might be restricted not only by pH but also by organic complexes of Al [187]. Another parameter limiting Al availability is the presence of silicic acid [200,201], instantly forming hydroxyaluminosilicates [202]. These findings suggest that silicic acid is a main control on ion concentrations in these soils.

**Figure 6:**
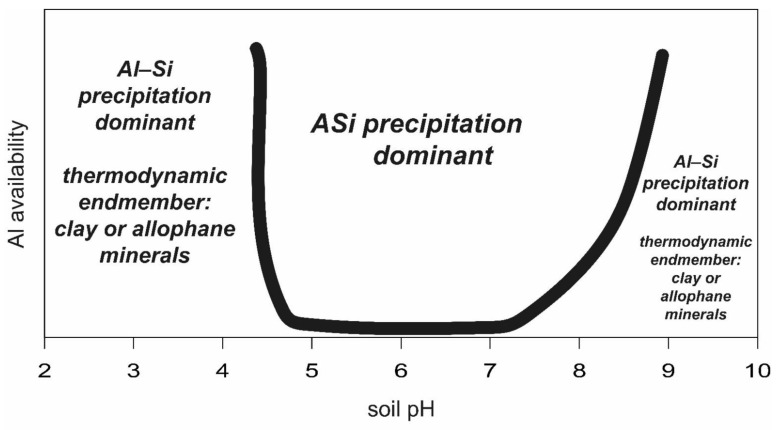
Predominance of ASi and Al–Si precipitations (with the thermodynamic endmembers clay or allophane minerals) in relation to soil Al concentrations and soil pH.

However, a quantification of the share of ASi in weathered volcanic soils is missing in most cases. In many publications, it is stated that allophane and imogolite are formed from volcanic ash soils favorable at pH > 5, referring mainly to Wada [192], who referred to Shoji et al. [196] and Shoji and Fujiwara [203]. The soils used in both studies were volcanic ash soils, and Shoji et al. [196] found large amounts of “opaline silica”—up to 8% in the “clay” sized fraction of the humus horizon—and referred it to ASi. However, Shoji et al. [196] did not show data for allophane or imogolite formation; instead, they determined their presence from occurrence patterns found for volcanic ash soils over a certain pH range. Shoji and Fujiwara [203] assumed that young andosols, especially, were characterized by a large amount of ASi, which was not determined by their analysis. This study again assumes the formation of allophane and imogolite in volcanic ash soils over a certain soil pH range from occurrence pattern over a certain soil pH range. It seems to be rather plausible from the view of aluminium (Al) availability (being low, between pH ~5 and ~7) [204] that allophane and imogolite can be found in high amounts in andosols at this pH range because these minerals are mostly stable under these conditions. The formation of these minerals is suggested rather to occur: (i) at the initial state of volcanic ash soils (because volcanic ash is alkaline and Al is mobile under alkaline conditions); and (ii) under acidic soil conditions (because Al is also mobile under pH lower than ~5) [204,205]. It has been shown that, at a until soil pH of about >3, allophane mineral dissolution leads to substantial formation of ASi [206]. Only at soil pH values lower than pH 3 can enough Al be mobilized such that allophane formation becomes possible [206]. This is in line with older findings, showing that the required Al concentration rapidly declines from pH 4 to pH 5 [207]. If the Al concentration in soil solution is too low, polymerized ASi will precipitate [206]. From a geochemical prospective, it can be assumed that the predominance of ASi precipitates over allophane/imogolite precipitates will prevail under increasing pH until the mobility of Al is strongly increasing (pH of ~7), changing the Si:Al stoichiometry to values of at least 1:1 for precipitates. Under common soil pH of andosols (with occurrence of allophane and imogolite) in the range between ~5 and ~7, the maximum Si concentration in pure water is ~100 ppm silicic acid (~30 mg/L Si). This value might be lower or even higher in soil solutions due to the interference with dissolved ions. The Al solubility in the pH range between ~5 and ~7 is ~1 mg L [185,204], which might result in favorable ASi formation in the soils, potentially explaining the occurrence of the high share of ASi found in volcanic ash soils. This high Si and low Al availability in the pH range between 5 and 7 was also found in a 2020 publication [208], showing a predominance of “opaline silica” (ASi) for this pH range (Figure 6). At higher pH values (pH ~9), allophane formation will occur [209] in the same way as under very low pH (see above) due to higher aluminum mobility. In addition, Mizota and Wada [210] suggested allophane formation after the removal of soluble Si by plant roots. However, in soil micro environments, the conditions can be far away from those in bulk soil, potentially leading to relatively strong differences in corresponding Al mobility [211].

Overall, the above-mentioned literature suggests that ASi is a dominant fraction of andosols/andisols. This high share of ASi might at least partly explain why andosols/andisols exhibit such a high water-holding capacity and nutrient availability (especially non-allophane andosols), properties which have been attributed to ASi and silicic acid originating from ASi [127,212,213] (see below). Only under low or high (<5 and >7) soil pH of andosols/andisols can a high concentration of Al in soil pore water readily leading to a formation of allophane be assumed [207,214], which suggests a minor role of ASi at low soil pH. Consequently, ASi might be a major component of andosols/andisols, and under some conditions even dominant, suggesting the ecological importance of ASi in these soils. Finally, Lowe [187] stated that the concentration of silicic acid is a major control on clay formation, with low silicic acid concentrations favoring “clay formation”. However, from the thermodynamic perspective, an instant formation of phyllosilicates from solution without condensation nuclei is impossible (Figure 6). Nevertheless, existing clay minerals can “grow” in certain soils (Figure 5). Clay minerals again represent thermodynamic endmembers, which are formed over months, decades, or millennia, via metastable phases starting from amorphous structures [215]. Amorphous silica is characterized by low interfacial free energy, and will consequently nucleate easily when soil solution exceeds the solubility of ASi [33]. For example, the clay mineral formation in Amazon shelf sediments needs between 12 to 36 months [216]. Soil conditions promoting the dissolution of one mineral might increase precipitation of another mineral (Figure 6).

However, the stability of minerals in soils might change by season. A good overview of seasonal changes in mineral stability is given by Zabowski and Ugolini [217]. Here, we will focus on the processes in the E horizon depending on the season as described in their study, because in this horizon, the most pronounced effects were found. The authors showed that mineral dissolution is dominant in spring, whereas in the summer minerals are rather stable. In autumn and winter, the stability of clay minerals depends on the specific mineral. The main factor for mineral stability in the analyzed soil was the high concentration of silicic acid (H_4_SiO_4_) in the soil pore waters. It is important to note that the soil used in this study was a tephra-rich soil, with a pH of about 4 in soil solution. The Al concentration in the soil pore water was only ~50% of the Si concentration during summer [218]. Hence, it is not surprising that ASi precipitations were found (in the form of opaline silica) as stable phases in summer [219], because in summer the limited precipitation in combination with high soil water loss due to evapotranspiration leads to a potential oversaturation of solutes and eventually to ASi precipitation. Conditions with high Si concentrations but low Al concentrations are well known from coastal sediments, where ASi can precipitate in high amounts as so called opal-Si [220]. High shares of opal-ASi were also found in permafrost soils, sometimes together with high (27%) contents of magnesium [221]. If elements such as K, Ca or Mg are available in soil solution in high concentrations together with silicic acid, different minerals might grow, on the edges of existing clay minerals, e.g., palygorskite (under high co-availability of silica and Mg) [222].

**Figure 7:**
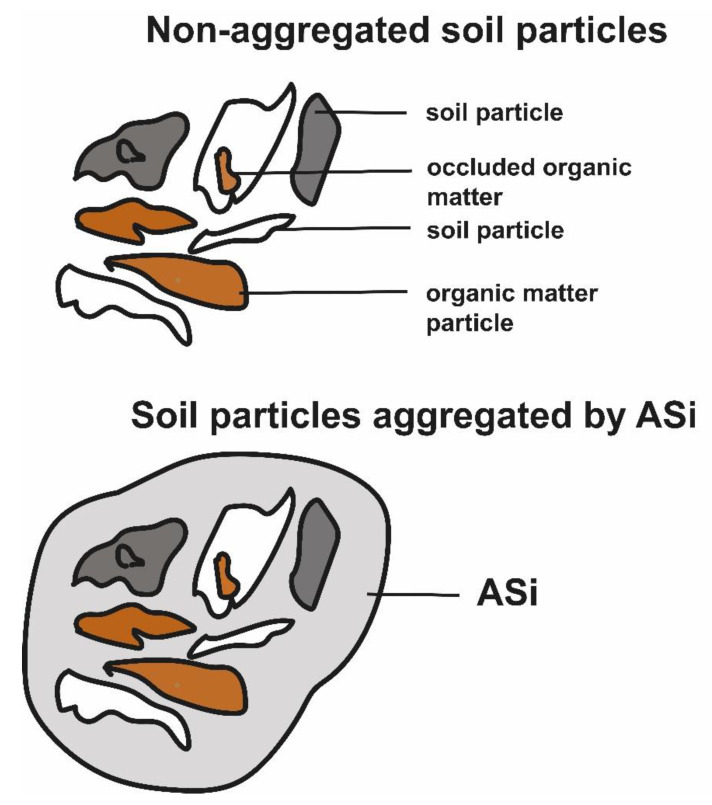
Conceptual scheme of soil particle aggregation by ASi precipitation.

The interdependency between the concentration of Si (or other main elements/compounds) in soil pore water and the subsequent potential precipitation of ASi or Al–Si precipitates might prevail the micro aggregate stability in soils. Micro aggregate stability in soils is the fundamental soil property that controls the resistance of soils against erosion and degradation [223,224]. Kemper and Rosenau [225] suggest ASi precipitation as an important mechanism that forces the cementing of soil particles [226], resulting in stable micro aggregates. If ASi is really that important for soil micro aggregate stability, the above-mentioned seasonal ASi cycling (with ASi dissolution in spring and stability in summer) might cause a strong effect on seasonal changes in soil micro aggregate stability. Secondary quartz was also found to glue soil particles together (e.g., duripan) [15,16]. Si infillings were shown several times by micro-morphological analyses [227,228,229]. Hence, ASi precipitations, as well as Si infillings, seem to be of high importance for micro aggregate stability in soils (Figure 7).

Analyses of neoformed material of the clay-sized mineral fractions from a river floodplain revealed particles composed of silica (>90%) or very small opaline silica (<0.1 µm) [230], similar to those once described by Drees et al. [15]. Intensive evapotranspiration [230] and freezing [193] may increase silicic acid concentrations potentially above saturation in the soil pore water, resulting in the formation of ASi precipitates. Besides the precipitation of ASi, silicic acid in soil solution might also bind to iron phases, for example [14]. Of note, ASi precipitates have been suggested to remove vast quantities of silicic acid from river waters in the Okavango delta [231].

ASi precipitation may comprise a large share of “clay neoformation”, and not only the (neo) formation of clay minerals. However, from a thermodynamic perspective, the instant formation of clay minerals from solution without condensation nuclei is impossible. Again, clay minerals represent thermodynamic endmembers. Clay minerals are formed over months, decades, or millennia, from metastable phases starting from amorphous structures. In a pH range of ~5 to ~7, the Al availability is several orders of magnitude lower compared to the availability of silicic acid, suggesting a restriction of clay mineral formation in this pH range. Increased solute concentrations driven by water losses due to evapotranspiration (especially in the summer) or freezing of the soil solution (winter) might result in pronounced ASi precipitation. ASi precipitation is suggested to be a main control of ion concentrations in soils. Consequently, the dominance of ASi precipitation is suggested to play a dominant role for soil micro aggregate stability, binding soil particles together.

## 6. Human Impacts and Global Change Effects on Soil Si Cycling

### 6.1. Si Availability Depending on Soil pH in Forests, Pastures, and Arable Crop Fields

**Figure 8:**
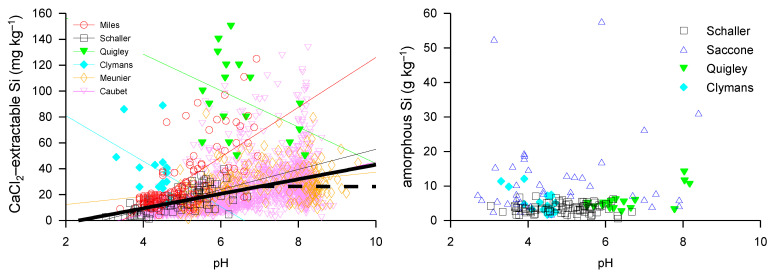
Si availability (CaCl_2_-extractable Si) in relation to soil pH (**left**). Data of more than 2500 soil samples from sugarcane production in South Africa [90,234], tropical rainforest soils [235], grasslands in the Serengeti [236], different ecosystems (continuous forest, grazed forest, pasture, and arable land, all southern Sweden) [233], rice paddy soils from South India [237], and soil from France [238] were analyzed. Amorphous Si in relation to soil pH (**right**). Data again from refs [233,235,236], and additionally from Hubbard Brook Experimental Forest, German forests, the Great Plains belt in northeastern/central Colorado and Kansas, and grasslands and forests were analyzed [53].

Si cycling ultimately primarily depends on the parent material in terms of mineral composition, with every mineral exhibiting different Si dissolution rates affecting Si availability [103]. The Si availability of soils is highly heterogenic at the landscape level [232], which might be due to hydrological effects. However, only a few articles show a larger dataset for Si availability in soils. To obtain a more comprehensive picture on how Si is affected by different human soil management techniques, we analyzed several articles with large datasets for available Si (CaCl_2_-extractable Si) and ASi (Figure 8). The gained dataset comprised more than 2500 data points for available Si (CaCl_2_-extractable Si) in relation to soils pH. We found an overall trend of increasing available Si (CaCl_2_-extractable Si) with increasing soil pH (Pearson’s *r* 0.42; *p* < 0.001) (bold black line, Figure 8, left). However, at a soil pH of ~7 and above, the concentration of CaCl_2_-extractable Si seemed to be more or less constant (dashed bold black line, Figure 8, left). We found no significant interdependency between soil ASi and pH, but non-agricultural soils generally showed higher ASi concentrations compared to agricultural soils [233].

Soils from sugarcane fields in South Africa showed an increasing Si availability (CaCl_2_-extractable Si), starting with ~10 mg Si kg^−1^ at soil pH between 3 and 4 (Pearson’s *r* 0.7; *p* < 0.001, red line, Figure 8, left), and highest values of below 20 to more than 120 mg Si kg^−1^ at soil pH between 6 and 7 [234]. Tropical rainforest soils showed a lower Si availability, with values between 1 and 10 mg Si kg^−1^ at soil pH between 3 and 4 and values between 3 and 40 mg Si kg^−1^ at soil pH between 5 and 7 (Pearson’s *r* 0.02; n.s., black dotted line, Figure 8, left) [235]. The lower Si availability was probably due to intense weathering in this region. Higher values for available Si were found in a study of grasslands in the Serengeti ecosystem in northern Tanzania, with values between 50 and 150 mg Si kg^−1^ at soil pH between 5.5 and 8.2 (Pearson’s *r* 0.71; *p* < 0.001, green line, Figure 8, left) [236]. Another study of available Si in soils of different ecosystems (continuous forest, grazed forest, pasture, and arable land) in southern Sweden showed the highest Si availability in continuous forest (43 to 89 mg Si kg^−1^ at soil pH between 3.3 and 4.5), and the lowest Si availability in arable land (13 to 29 mg Si kg^−1^ at soil pH between 4.6 and 4.7) (Pearson’s *r* −0.69; *p* < 0.001, cyan line, Figure 8, left) [233]. For rice paddy soils, Meunier et al. [237] found an increasing Si availability from soil pH of 4.8 to 9.3 in the range of 1 to 83 mg Si kg^−1^ (Pearson’s *r* 0.29; *p* < 0.001, orange line, Figure 8, left). The large dataset of Caubet et al. (~2000 soil samples from France) on soil CaCl_2_-extractable Si (Pearson’s *r* 0.53; *p* < 0.001, purple line, Figure 8, left), published most recently, also showed an increase in CaCl_2_-extractable Si, with soil pH starting from pH of 3.5 with values of ~2 mg Si kg^−1^ to pH of 7 (range between 2 to more than 100 mg Si kg^−1^) [238]. However, from pH 7 and above, the CaCl_2_-extractable Si was more or less constant (values from 0 to more than 135 mg Si kg^−1^) and seemed to have nearly no dependency on the increasing soil pH. However, this study found a positive relationship of CaCl_2_-extractable Si with soil Fe oxides and organic carbon content [238]. Looking more closely at the relationship between soil pH and CaCl_2_-extractable Si, the data without the information from Caubet et al. [238] showed the same pattern as the dataset with data from Caubet et al. [238], with an increase in CaCl_2_-extractable Si until soil pH of 7, and afterwards more or less constant values for CaCl_2_-extractable Si at pH values higher than 7. Based on a the large dataset, containing data resulting from all of the studies mentioned above, we propose a linear relationship for the interdependency between soil pH and CaCl_2_-extractable Si, which is contrast to Haynes, who assumed a more complex relationship [90,234].

However, because the datasets of Haynes [90,234] are included in the large dataset, we suggest that some outliers in their datasets or specific properties of the tested soils (probably resulting from agricultural practice in sugarcane production) might cause this difference. Moreover, the negative relationship between CaCl_2_-extractable Si and soil pH suggested by Quickley and Clymans (Figure 8, left) may be of stochastic nature due to the small size of their datasets.

### 6.2. Amorphous Silica Contents in Forests, Pastures, and Arable Crop Fields

The ASi concentration in soils is, in most cases, below 15 g Si kg^−1^ for soil pH values from 2.5 to 8.3 (Figure 8, right). Only in soils of non-agricultural sites can higher ASi values of up to 55 g Si kg^−1^ be found [53] (Figure 8). In another study, forest soils showed a distinct pattern of soil ASi concentrations, with highest values (~25 g kg^−1^) in organic soil horizons, ~14 g kg^−1^ in A-horizons, and between 3 and 6 g kg^−1^ in deeper horizons [239]. In general, soils used for agriculture (crop production) exhibit lower concentrations of ASi compared to forest or pasture soils. This might result from the yearly Si exports by crop harvest (export of Si in grain and/or straw) [101,240], leading to a decrease in the ASi pool in soils, because many crops are Si-accumulating plants [90]. This is underpinned by the results of a long-term experiment (more than 150 years), showing that straw removal decreased soil ASi concentrations [241]. Grassland degradation (decreasing biomass per m^2^) was shown to lead to a strong decrease in plant-available Si [242], potentially due to a disturbed Si cycle characterized by a decreased Si uptake by plants and decreased Si recycling via litterfall and litter decomposition. For rice paddy fields, a yearly Si export of about 150 kg Si ha^−1^ year^−1^ by crop harvest was found [243]. The yearly Si export is dependent on whether only grain or grain and straw is removed from the field [240], with grain exports of 4–5 kg Si ha^−1^ year^−1^ for corn and ~120 kg Si ha^−1^ year^−1^ for the removal of total aboveground biomass. The Si export for wheat is between 37 and 110 kg Si ha^−1^ year^−1^, depending on whether only grain or grain and straw is removed [240].

The highest concentration of ASi in soils, i.e., up to 8% (80 g Si kg^−1^), was found in volcanic ash soils [196] (see Section 5). It was also shown that pedogenic ASi dissolution seems to be more affected by soil pH than biogenic ASi dissolution [244]. Furthermore, it is well known that climax forests are characterized by enhanced mineral weathering, Si uptake and accumulation by plants, and Si recycling via litterfall and litter decomposition [93]. The Si uptake rates of climax forests are in the range of Si-mobilization from weathering [93]. A change of the dominating vegetation by humans (deforestation) is leading to a large Si export, declining the soil ASi pool [93]. In addition, disturbances such as increased erosion [93] or human-caused fires [245] have the potential to alter ASi pools and Si availability in soils [246]. Human-caused fires are used in many regions of the world to increase nutrient availability [247,248], which potentially also increases Si availability in soils [249].

### 6.3. Si Fertilization

The use of Si as fertilizer started in the 1950s in Japan, and is a common contemporary practice in many parts of the world [250]. Most Si applications in agriculture are done for pest control or to increase the beneficial effects of Si on plant performance and yield [251]. In rice cultivation systems, farmers use about 900 kg Si ha^−1^ or more (up to 3000 kg Si ha^−1^) per year to increase yields [252], because Si is important for rice production [253]. The most applied Si fertilizers are wollastonite, silica gel, liquid suspensions or solutions of meta-silicate, silicic acid, potassium-silicate, steel slag, fly ash, and blast furnace slag, but rice and *Miscanthus* straw or even manure are also used [100,250]. The application of these materials increases Si availability in soils, potentially changing soil Si cycling for decades [250]. It has been shown that Si fertilization using ASi-rich biochar leads to an increase in plant-available Si depending on soil type (plant-available Si was found to be higher in soils with lower soil buffering capacity), accompanied by a stronger soil pH increase due to biochar application [168]. Si fertilization might change the proportion of silicic acid polymerization and de-polymerization, because fertilization is potentially increasing silicic acid concentrations in soil solution (see above). The different species of silicic acid potentially affect the binding affinity of silicic acid to soil minerals, and thus compete with other compounds such as phosphates or organic matter for binding sites on soil minerals (see below). Thus, on the one hand Si availability in soils is decreased by removing Si every year by crop harvest and increased soil erosion caused by land use, while on the other hand Si availability is increased by human-caused fires or the application of Si-rich fertilizers.

Moreover, humans might alter the soil Si cycle in the future by facilitating global change. In this context, global soil Si availability might increase because of the predicted temperature increase due to global warming, which potentially enhances mineral weathering rates [254,255], and thus silicic acid release [6]. In addition, more frequent and longer drought periods are predicted on continental (e.g., European) and global scales due to climate change [256,257]. Soil drying in turn can lead to ASi precipitation [219], driven by an oversaturation of solutes in dry soils (see above). Hence, the predicted increase in drought periods might lead to an increase in soil ASi precipitates. However, it might also be possible that severe droughts and resultant decreased soil water contents might cause decreased weathering rates [258], which in turn might reduce the mobilization of elements such as Si. Additionally, droughts might lead to a decreased silicic acid uptake by vegetation, because Si uptake is linked to water uptake [259]. Presently, it remains unclear which of these scenarios is more likely, and more data are urgently needed for reliable predictions.

Si availability in soils is increasing with increasing soil pH. Amorphous Si concentrations in soils are considerably lower in agricultural soils compared to non-agricultural soils, such as forest or steppe soils. Agriculture is strongly decreasing Si availability in soils by removing Si every year through crop harvest accompanied by increased soil erosion. However, some agricultural practices might also increase Si availability in soils, e.g., human-caused fires (mobilizing unavailable Si) or the application of Si-rich fertilizers. At present, the future impact of global change on the Si cycle in soils remains unpredictable, because sufficient and reliable data are still missing in this field.

## 7. Importance of Si for Crop Production

In this section, we will give a short overview of the importance of Si as a beneficial element for crop production. A comprehensive review on Si and its role in plant performance is given by Katz et al. [260] in this Special Issue.

### 7.1. The Need of Crops for Si

It has been estimated that rice plants accumulate ~270 kg Si ha^−1^ year^−1^ [243]. Si accumulation seems to be directly, positively linked to yields in rice growing systems [136,261,262]. In a laboratory study, it was clearly shown that a reduction in Si accumulation leads to a strong decrease in rice yields [263]. The positive effects of Si on crop yields were also shown for sugarcane [137,264] and wheat [29]. The Si accumulation in crops was estimated by Tubana et al. [100] to be 99 kg Si ha^−1^ year^−1^ for barley, 129 kg Si ha^−1^ year^−1^ for maize, 48 kg Si ha^−1^ year^−1^ for oat, 329 kg Si ha^−1^ year^−1^ for rice, 62 kg Si ha^−1^ year^−1^ for sorghum, 59 kg Si ha^−1^ year^−1^ for soybeans, 1408 kg Si ha^−1^ year^−1^ for sugar beet, 160 kg Si ha^−1^ year^−1^ for sugarcane, and 108 kg Si ha^−1^ year^−1^ for wheat.

### 7.2. The Importance of Si for Mitigating Abiotic Stress

Accumulation of Si in plant biomass has many positive effects on plant performance, such as enhanced nutrition, pest control, and protection against abiotic stress (e.g., metal toxicity, ultraviolet radiation, and drought). The increase in biomass production and yields by Si fertilization might be explained by increased plant nutrition [265,266,267] and nutrient use efficiency [29]. Furthermore, silicic acid released from Si fertilizers competes with nutrients (e.g., phosphorus) for binding sites at soil minerals, causing nutrient mobilization [126,127,268]. Large amounts of phosphorus in soils are unavailable for plants [269,270]; therefore, Si fertilization could be used to reduce the need for phosphorus fertilizer application due to the mobilization of unavailable phosphorus by silicic acid.

Silicon has been suggested to mitigate drought stress for crop plants [25,271,272,273,274]. In their review, Zhu and Gong [266] suggested reduced transpiration to be one of the main mechanisms for enhanced drought resistance induced by Si. However, the data available in the literature are inconsistent. While some studies showed a positive effect of Si with increased transpiration rates [275], others found a decrease in transpiration rates during droughts (summarized by Zhu and Gong [273] and Rizwan et al. [274]). However, drought stress mitigation by Si application might not only result from changes in plant performance, but also from soil–water relationships. There is some literature claiming that amorphous silica (ASi) is capable of increasing the water-holding capacity of soils [12], in some cases dramatically [212,276]. This increase in water-holding capacity by ASi might be explained by a silica gel formation from polysilicic acid or colloidal amorphous silica, in the pH range of 4 to 7 with a maximum at pH 5 [277]. Most recently, amorphous silica has been shown to strongly increase the water-holding capacity and plant-available water in soils [213]. It was shown that an increase in ASi by 1% or 5% (by weight) increased plant-available water by up to >40% or >60%, respectively [213]. However, a comprehensive picture of the effect of ASi contents on the water-holding capacity of soils is still missing. It is currently also not clear whether the effect of Si on soil water-holding capacity and plant-available water or on plant physiology is the more important one. Furthermore, it is not clear how these effects are linked or interact.

A study by Goto et al. [278] showed that Si accumulation in above-ground plant tissues can reduce stress from ultraviolet radiation. It was suggested that the formation of a pronounced double Si layer in the near-epidermis region might act as a filter against ultraviolet radiation [27]. Furthermore, Si was shown to mitigate salt stress [273,274,279] and the toxicity of metals and metalloids [280]. The mitigating effect of Si in terms of decreasing toxicant uptake might be caused by direct competition of silicic acid with, e.g., arsenic (in the form of arsenite) for plant uptake, because both compounds are taken up via the same uptake systems [281]. Moreover, the plant uptake of toxic iron or manganese species also seems to be suppressed by Si [282,283].

### 7.3. The Importance of Si for Mitigating Biotic Stress

High Si concentrations in plant biomass can act as a defense against herbivores (mammals and insects) [30,284,285]. Si compounds in biomass (phytoliths) might reduce the palatability and digestibility of plant materials, and thus reduce herbivores’ feeding preferences [286]. Consequently, Si accumulation in plant biomass might lead to higher biomass production and yield under high pressure from herbivores. In addition, fungal diseases in rice plants were decreased by plant Si accumulation, as found in several studies [31,287,288]. It was shown that this effect might be mainly due to a Si layer in the mesophyll cells [289].

The management of Si availability seems to be a very promising tool to increase, or at least maintain, crop production. Si accumulation was shown to increase plant nutrition, nutrient use efficiency, biomass production, and yield, especially under stress conditions. Enhanced Si accumulation in plants was shown to reduce nutrient imbalances, drought stress, salt stress, stress from toxic metals and metalloids, and stress from ultraviolet radiation. However, not only was abiotic stress found to be mitigated by Si accumulation, but biotic stress was as well (i.e., herbivory and fungal diseases). Overall, increasing Si availability in soils, and thus Si accumulation in plants, seems to be a promising tool to reduce the need of farmers for common fertilizers (silicic acid can mobilize nutrients in soils) and pesticide application (Si accumulation in plants can reduce their susceptibility to pests) [290].

## 8. Outlook

The picture for the soil Si cycle, availability, and function is currently patchy, rendering an overall conclusion for the importance of Si for ecosystem functioning impossible. For a better understanding of the Si cycle, Si availability, and function, we need more research disentangling the interdependency between silicic acid polymerization/depolymerization and Si precipitation in relation to concentrations of ions and anions in soil solutions. Furthermore, we need studies investigating the importance of ASi precipitation for soil micro aggregate stability in more detail. Regarding bASi, more information on protistic bASi (testate amoebae, diatoms) in terrestrial ecosystems is urgently needed. The outcomes of such research will help us to gain a more comprehensive picture of the role of biota, biosilicification, and bASi pools in Si cycles of terrestrial ecosystems, a picture which is still very limited (cf. Figure 2). Furthermore, the phytogenic Si continuum should be in the focus of interdisciplinary research. The importance of ASi should gain more attention regarding soil fertility, resistance against erosion, main soil components, diagenesis, soil water-holding capacity, as well as changes in soil processes caused by global change.

Additional care becomes necessary when interpreting results in the light of thermodynamics, because the reaction time of Si species is much lower than that of many other soil components (months to millennia). Finally, we need to understand the implications of changes in polymerization/depolymerization/precipitation of silicic acid for the availability and cycling of Si and nutrients, as well as on soil water storage, ecosystem productivity, and ecosystem services. To attain an overall picture of Si effects at the ecosystem level and to unravel the interplay between abiotic and biotic factors in Si cycling, the obtained knowledge should be included in systematic approaches integrating soil science and ecological research, as well as plant, animal, and microbial physiology.

## Figures and Tables

**Table 1:** Most important extraction procedures and Si fraction supposed to be extracted.

Extractant	Procedure (Soil: Extractant Ratio/Extraction Time/Temperature	Si Fraction Supposed to Be Extracted	References
H_2_O	10 g: 50 mL/21 days/room temperature	Water-soluble Si	[37]
H_2_O	10 g: 100 mL/4 h/room temperature	Water-soluble Si	[35,36]
0.01 M CaCl_2_	1 g: 20 mL/16 h/room temperature	Readily available + above listed fractions	[40]
0.01 M CaCl_2_	10 g: 100 mL/1 h/room temperature	Readily available + above listed fractions	[41]
0.5 M NH_4_-acetate; adjusted to pH 4.8	1 g: 10 mL/1 h/room temperature	Soluble and some exchangeable Si + above listed fractions	[35]
0.5 M acetic acid	1 g: 10 mL/1 h at room temperature//--resting	Soluble and some exchangeable Si + above listed fractions	[42]
NH_4_ citrate	10 g: 25 mL/80 h/room temperature	Soluble, exchangeable and specifically adsorbed Si + above listed fractions	[43]
0.2 M NH_4_ oxalate; adjusted to pH 3.0	2 g: 100 mL/1 h room temperature//dark room	Si bound in amorphous and poorly crystalline pedogenic oxides + above listed fractions	[47]
Mehlich-III solution at pH 2	2 g: 42 mL/5 min/room temperature	Si bound in amorphous and poorly crystalline pedogenic oxides + above listed fractions potential share of crystalline Si is smaller	[44,45]
0.5 M NaOH	soil: extractant ratio of less than 100 mg: 100 mL/2.5 min/boiling	Si from allophanes and amorphous silica + above listed fractions	[48]
0.2 M NaOH	1 g: 400 mL/5 h, ~120–168 h/room temperature	Amorphous silica and low amounts of crystalline Si + above listed fractions	[49]
0.1 M Na_2_CO_3_	30 mg: 40 mL/5 h/85 °C/aliquots after 2, 4 and 5 h/	Biogenic silica in sediments and water + above listed fractions	[51,52]
0.1 M Tiron, pH 10.5	25 mg: 30 mL/1 h/80 °C	Si bound in amorphous silica in soils + above listed fractions	[56]
Sequential extraction			[57]
**Step 1**: 0.01 M CaCl_2_	1 g: 5 mL/TIME/rinsed with pure water room temperature/	Readily plant-available Si	
**Step 2**: 0.01 M acetic acid	soil solution ratio = 1:10/24 h/room temperature/	Adsorbed Si fraction	
**Step 3**: H_2_O_2 (_17.5% in water)	soil solution ratio = 1:20/24 h/plus 10 mL 35% H_2_O_2_/85 °C until reaction is complete	Si bound to organic matter	
**Step 4**: 0.2 M NH_4_ oxalate and 0.2 M oxalic acid	1:50/8 h/overnight treatment with UV light/room temperature/	Si occluded in pedogenic (hydr)oxides	
**Step 5a**: sodium polytungstate 0.2 M NaOH solution	50% residue of step 4 pre-preparation with sodium polytungstate, afterwards soil solution ratio = 1:400/168 h/room temperature/	Biogenic ASi	
**Step 5b**: 0.2 M NaOH solution	Other 50% residue of step 4 soil solution ratio = 1:400/168 h/room temperature	Total ASi, also calculate minerogenic ASi (total ASi—biogenic ASi)	
